# Factors Associated with Adverse Reactions to BNT162b2 COVID-19 Vaccine in a Cohort of 3969 Hospital Workers

**DOI:** 10.3390/vaccines10010015

**Published:** 2021-12-23

**Authors:** Mario Rivera-Izquierdo, Eva Soler-Iborte, Javier Pérez de Rojas, María Dolores Pegalajar-García, Ana Gil-Villalba, Ricardo Ruiz-Villaverde, María del Carmen Valero-Ubierna

**Affiliations:** 1 Service of Preventive Medicine and Public Health, Hospital Universitario San Cecilio, 18016 Granada, Spain; eva.soler.sspa@juntadeandalucia.es (E.S.-I.); javier.perez.rojas.sspa@juntadeandalucia.es (J.P.d.R.); mariac.valero.sspa@juntadeandalucia.es (M.d.C.V.-U.); 2 Department of Preventive Medicine and Public Health, Hospital Universitario San Cecilio, 18016 Granada, Spain; 3 Instituto Biosanitario de Granada (ibs.GRANADA), 18012 Granada, Spain; ricardo.ruiz.villaverde.sspa@juntadeandalucia.es; 4 Service of Dermatology, Hospital Universitario San Cecilio, 18016 Granada, Spain; mariad.pegalajar.sspa@juntadeandalucia.es (M.D.P.-G.); ana.gil.villalba.sspa@juntadeandalucia.es (A.G.-V.)

**Keywords:** vaccination, COVID-19, adverse events, reactions, symptoms, healthcare, professionals, Comirnaty, Pfizer

## Abstract

Factors associated with adverse reactions to BNT162b2 COVID-19 vaccine reported by hospital workers are unclear. Our aim was to collect all reported adverse events in a cohort of hospital workers and to analyze the factors associated with their presence. We conducted an observational longitudinal study on all hospital workers of our center who received COVID-19 vaccination from 27 December 2020 to 1 September 2021. Information on adverse events was reported telephonically and confirmed through clinical records. Chi-square and *t* tests as well as multivariate logistic regression models were used. Cluster analysis was designed to explore associations between reactions. A total of 3969 hospital workers were included in the sample. Of the total sample, 182 workers (4.6%) reported adverse events. The most frequent symptoms were general malaise (*n* = 95), fever (*n* = 92), arthromyalgia (*n* = 80), and headache (*n* = 47). The factors associated with adverse events in adjusted analyses were an antecedent of COVID-19 infection (OR = 2.09, 95% CI: 1.47–2.98), female sex (OR = 1.51, 95% CI: 1.03–2.20), and professional category (OR for physicians = 0.41, 95% CI: 0.21–0.80). We report a low frequency of adverse events in hospital workers after COVID-19 vaccination and no severe reaction. Men and physicians underreported their symptoms. These data should guide future strategies for recording adverse events and future research on COVID-19 vaccination safety.

## 1. Introduction

The quick and effective development of vaccines against COVID-19 has played an important role in the control of the pandemic [1] and represents one of the most meritorious and relevant advances of the history of science [2]. As of October 2021, the European Medicines Agency (EMA) has authorized the use of four vaccines against COVID-19 in Spain: BNT162b2 (Comirnaty^®^), mRNA-1273 (Moderna^®^), AZD1222 (Vaxzevria^®^), and Ad26.COV2.s (Janssen^®^). Although all these vaccines have proven their efficacy and safety, several studies during 2021 have reported adverse events in clinical series and post-authorization trials [3,4,5].

The first vaccine that was implemented in Spain on 27 December 2021 was BNT162b2. Following the recommendations of the Spanish Ministry of Health’s Strategy on COVID-19 Vaccination [6], this vaccine was first implemented in the oldest population subgroup and in workers of health and sociosanitary centers. Therefore, all hospital workers in Spain were offered BNT162b2 vaccination in a double-dose regimen, separating the doses by at least 21 days.

Although the efficacy and safety of this vaccine have been widely studied [7,8], several articles have reported adverse reactions to this vaccine in the general population [9] and in healthcare workers [3,4,5]. To date, no study has evaluated these adverse events in hospital workers in large sample-sized cohort in Spain, and the factors associated with these events are not clear to date. Coggins et al. suggested that women were more susceptible to report these events [5], but this information contrasts with other studies that did not find any clear association.

However, correct communication in reporting adverse events after vaccination is crucial for obtaining unbiased information on the security and safety profile of new COVID-19 vaccines. In addition, data on the most frequent adverse reactions and associated risk factors in certain subgroups (e.g., hospital workers) seem necessary to provide more information and increase confidence in these populations in view of a third dose of the vaccine, which has already been implemented in certain contexts [6].

The objectives of this study were to collect all adverse reactions to BNT162b2 COVID-19 vaccine reported by all hospital workers of our center and to analyze the main factors associated with the reported adverse events.

## 2. Materials and Methods

### 2.1. Study Design

We conducted a longitudinal observational study on all workers of our center who received vaccination against COVID-19 through administration of BNT162b2 Comirnaty^®^ vaccine. The recruitment period started on 27 December 2020, when the first doses of the vaccine arrived at the hospital, and continued to 1 September 2021, the end of our follow-up. All the workers of our center received the same vaccine according to the same vaccination schedule, following the recommendations established by the Spanish Health Ministry for workers of health and sociosanitary centers during the follow-up period of the study [6]. The recommended regimen consisted in a double dose, including a minimum interval of 21 days between the two doses. The only contraindication to the vaccine during this period was the presence of COVID-19 or other infection at the time of vaccination or a severe adverse reaction to the first dose. The hospital workers acceded voluntarily to vaccination in a specific service enabled by the hospital and managed by the Service of Preventive Medicine and Public Health of our center, enhanced by other services and professionals during the higher-demand periods.

### 2.2. Data Source and Variables

We identified the registries collected by the Preventive Medicine and Public Health Service’s personnel for recruiting the hospital workers who received vaccination and for collecting the main sociodemographic variables (sex, age, and occupational category). Occupational category was divided into the following subcategories: physicians, healthcare resident students (training physicians, nurses, and pharmacists), nurses, auxiliary nurses, wardens, other healthcare workers (including physiotherapists, occupational therapists, pharmacists, psychologists, and health technicians such as laboratory technicians and radio-diagnostic technicians), administrative workers (including administrative personnel, management positions, and clinical documentation and admission technicians), and other non-healthcare workers (including security personnel, kitchen workers, maintenance technicians, and cleaning staff).

Data on COVID-19 vaccination, adverse reactions reported, history of COVID-19 infection, and serological tests were obtained from the clinical histories with the support of the hospital’s Information and Communication Technology (ICT) service. The variable “pain at the site of injection” was not considered as an adverse reaction but as a normal situation, given the high number of patients reporting this symptom in previous reports, which was over 70% [4,5]. Vaccination regimen (single-dosed or double-dosed) was recorded. Participants with previous COVID-19 infection were divided in three groups depending on the moment of the first positive PCR: infection before the first dose of the vaccine, infection during the vaccination period (including workers with a positive PCR from the day of the first vaccine dose to 7 days after the second dose), and infection after the vaccine (infection from 7 days after the second dose). These times were based on the immunization periods reported in the Comirnaty^®^ technical data sheet by the European Medicines Agency [10]. During the first vaccine consultation, all hospital workers included in the cohort received information on how to report adverse reactions or persistent symptoms after vaccination, and a specifical telephone number was enabled for this purpose. The members of the research team collected these data and included them in the medical records.

### 2.3. Analyses

First, descriptive univariate analysis was performed to characterize the sample. Means and standard deviations were calculated for quantitative variables, and absolute and relative frequencies were calculated for qualitative variables.

Second, bivariate analyses were conducted comparing the subgroup of patients who reported adverse reactions to the vaccine and the subgroup of patients who did not. Chi-square tests were used for comparing qualitative variables between the two groups, and *t* tests for comparing quantitative variables. When the conditions for application were not met, Fisher exact tests and Mann–Whitney tests were applied, respectively. The association between symptoms was graphically analyzed through a dendrogram for cluster analysis.

Third, multivariate logistic regression models were designed, and odds ratio (OR) for the development of adverse reactions were calculated. Models were adjusted for sex, age, antecedent of COVID-19, and vaccination doses.

All analyses were performed using Stata (StataCorp^®^, College Station, TX, USA), version 15.0.

### 2.4. Ethical Considerations

The requirements established by the Declaration of Helsinki for research with human data were met. The research team used an anonymized database for conducting the analyses. No potentially identifiable variables were used. No written informed consent was provided, as the only data used were from an anonymized database. The protocol of the study was approved by the Provincial Ethical Research Committee, Granada.

## 3. Results

### 3.1. Description of the Sample

The cohort involved a total a of 3969 hospital workers of our center who received vaccination against COVID-19 with BNT162b2 after voluntary agreement. This number represent over 95% of the total workers during the period of the study. Of them, 2902 (73.1%) were women, as usual for healthcare workers in our country, and the mean age was 46.4 years (standard deviation: 13.9), corresponding to that of the working population. The most frequent occupational categories were nurses (23.0%), auxiliary nurses (18.3%), other non-healthcare professionals (15.7%), physicians (13.7%), wardens (8.4%), and other healthcare professionals (8.3%). A total of 182 patients (4.6%) reported adverse reactions to vaccination. The distribution of the sociodemographic and clinical characteristics of the sample according to the report of adverse reactions is shown in Table 1.

The main variable associated with reporting adverse reactions was the antecedent of COVID-19 infection (*p* < 0.001). Specifically, the antecedent of COVID-19 infection previous to the vaccine (*p* < 0.001) was a risk factor for reporting adverse reactions or persistent symptoms after vaccination. Women also reported more frequently adverse reactions than men (*p* = 0.027). The professional category was also associated with the report of adverse reactions (*p* = 0.010). Concretely, physicians tended to report less reactions (*p* = 0.003), and administrative workers reported reactions more frequently (*p* = 0.002).

The single-dosed regimen was also associated with reporting reactions (*p* < 0.001), as the presence of adverse reactions made some workers refuse the second dose.

### 3.2. Specific Adverse Reactions

Table 2 shows the specific adverse reactions reported in our study (*n* = 182). The most frequent symptoms were general malaise (*n* = 95), fever (*n* = 92), arthromyalgia (*n* = 80), headache (*n* = 47), and nausea or vomiting (*n* = 26). Most of the hospital workers who reported any adverse reaction reported more than one symptom (mean, 2.5 symptoms).

The sum of individual adverse reactions (*n* = 451) is higher than the number of workers reporting adverse reactions (*n* = 182) because most of them reported more than one reaction simultaneously.

**Figure 1:**
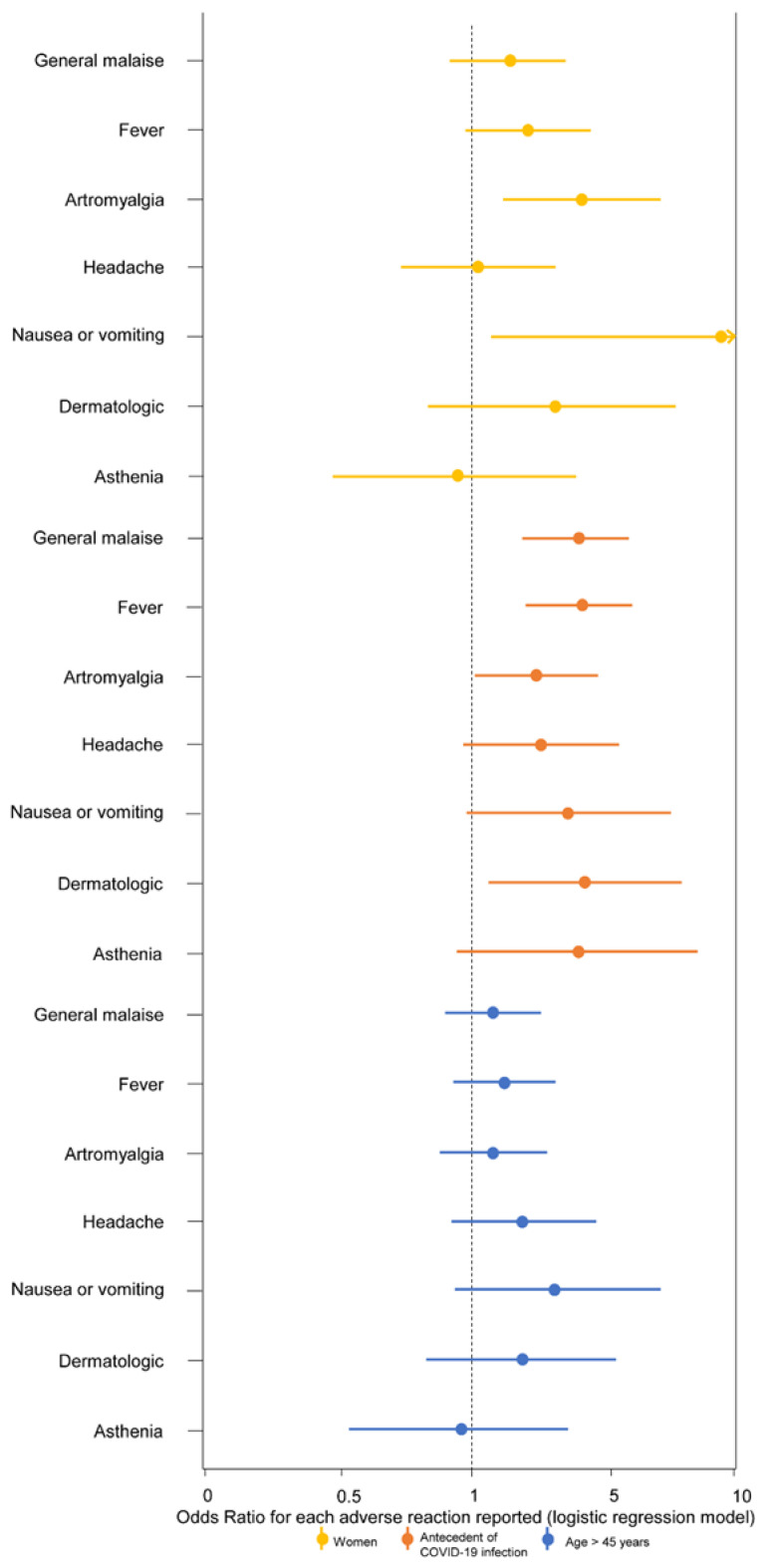
Associations of female sex, antecedent COVID-19 infection, and age >45 years with the most frequent adverse reactions after BNT162b2 vaccine in hospital workers. Odds Ratios are presented with 95% confidence interval for each outcome (specific symptom). The reference categories were men, no antecedent COVID-19 infection, and age ≤45 years, respectively.

**Figure 2:**
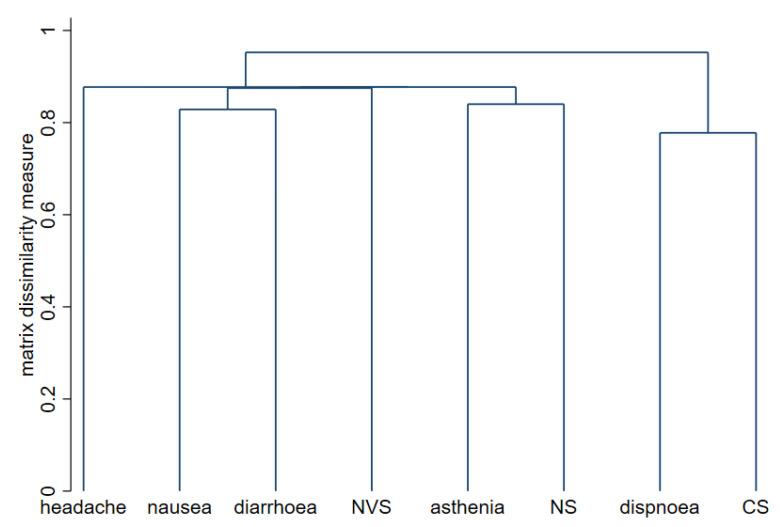
Dendrogram for cluster analysis of adverse reactions to BNT162b2 vaccine. The strongest relationships between the symptoms are marked by lower links on the y-axis. NVS, neurovegetative symptoms; NS, neurological symptoms; CS, catarrhal symptoms.

**Figure 3:**
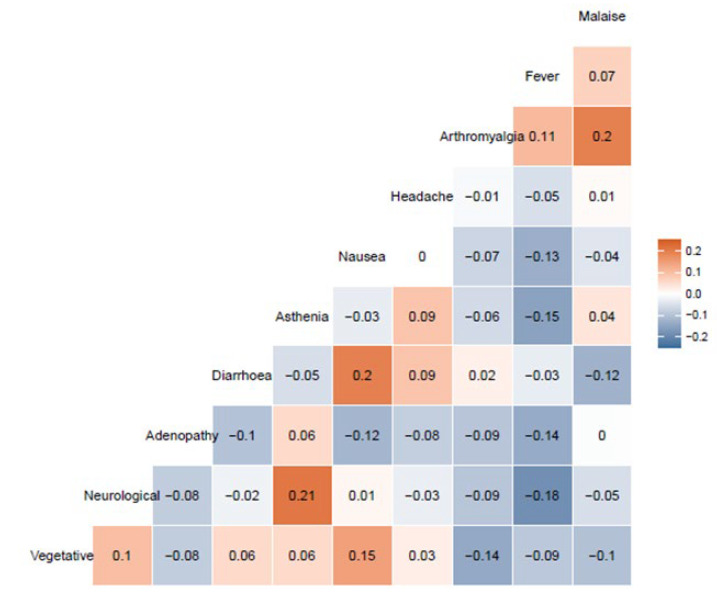
Correlation map of the associations between individual adverse reactions. Only the 10 most frequent reactions are represented. Malaise, general malaise; adenopathy, painful adenopathy; vegetative, neurovegetative symptoms.

The association of all the specific symptoms with female sex, antecedent COVID-19 infection, and age over 45 years is graphically shown in Figure 1. Briefly, women more often presented arthromyalgia and nausea or vomiting. Workers infected before vaccine showed a higher frequency of general malaise and fever, and age was not associated with any individual symptom. To explore the possible associations between symptoms in our cohort, cluster analysis was conducted. Figure 2 shows this association through a dendrogram. The most frequent associations were dyspnea and catarrhal symptoms; asthenia and non-severe neurological symptoms; nausea and diarrhea. Figure 3 shows a correlation map of the associations between individual adverse reactions.

Arthromyalgia and general malaise, nausea and diarrhea (both digestive symptoms), and neurological symptoms and asthenia were the only associations that showed a correlation R > 0.20. Neurovegetative symptoms and nausea also showed a correlation of R = 0.15, as nausea can also be part of a neurovegetative reaction. Finally, an inverse correlation was observed between neurological symptoms and fever (R = −0.18) and asthenia and fever (−0.15). In fact, the presence of fever was inversely correlated with the presence of the remaining symptoms, except for arthromyalgia. The other associations showed no relevant correlations (−0.15 < R < 0.15).

### 3.3. Multivariate Analysis

Table 3 shows the factors associated with the reported adverse reactions in crude and adjusted analyses. After adjusting for the main confounders in the multivariate analysis, female sex, vaccination regimen (single-dose), and antecedent COVID-19 infection remained as the main factors associated with the report of adverse reactions.

## 4. Discussion

We present the results of a study on a large cohort of hospital workers who received vaccination against COVID-19 with BNT162b2. Our study confirms the low frequency of adverse reactions after vaccination, and the wide variety of possible symptoms.

All reported reactions were non-severe, mainly general malaise, fever, arthromyalgia, and headache. These symptoms coincide with the normal reaction to other vaccines, such as trivalent Influenza vaccine [11,12], pneumococcal vaccines [12], or adenovirus vaccines against COVID-19 [13]. We also showed a correlation between several symptoms, as presented in Figure 3. Interestingly, fever was inversely associated with the presence of other symptoms. This fact might be explained by the intensity and relevance of fever, which might hide the report of other potential minor symptoms.

According to the technical datasheet of Comirnaty^®^ from the European Medicine Agency [10], the most frequent reactions in the population over 16 years of age were local pain, fatigue, headache, and myalgia. We present the results of hospital workers (18 to 65 years old), who tended to report a higher frequency of fever and arthromyalgia and a lower frequency of fatigue. Local pain was not considered as a reaction to the vaccine in this study, as we considered this symptom as normal after any vaccination or injection, and several series reported a frequency of this symptom from 70% to 91% after BNT162b2 vaccination [4,5]. We broaden the existing information by proving risk factors for reporting these symptoms. We found that women, administrative workers, and workers who had been infected by COVID-19 tended to report more reactions to the vaccine. It is possible that a previous infection, the readiness of the immune response, and the availability of antibodies increase the susceptibility to vaccine reaction. Therefore, non-severe adverse reactions should not be necessarily negative, as a causal relationship between immunological responses and adverse reactions has been reported [14]. Nevertheless, our results suggest that men (specially physicians) tend to underreport their symptoms after vaccination, thus biasing the results of pharmacovigilance studies. Communicative strategies should be reinforced in this risky groups identified in our study in order to raise their awareness to report symptoms. The association with the single-dosed regimen is unvaluable, as many of the workers who received a single dose refused to have a second dose because of adverse events; therefore, a reverse casual bias is highly probable (the reaction is associated with the subsequent regimen, and not the other way around). It is important to note that the distribution of the sociodemographic characteristics in this study coincides with the population of healthcare workers in Spain and could not match populations of other countries. For instance, the frequency of women (73.1%) in our study is similar to that reported by the National Institute of Statistics [15] in 2020 (52.2% of physicians and 84.1% of nurses are women).

This study has some limits. First, it was a one-center study. However, we included over 95% of the hospital workers, obtaining a highly representative sample. Nevertheless, the external validity of the results is limited by the type of population (hospital workers) and the country (Spain). Second, we collected only reported adverse reactions by phone. It is probable that these data are underestimated, as many workers with less severe symptoms may have not reported them. Finally, data on previous health conditions in the participants, which could affect the presence of adverse reactions, were not collected. Therefore, the associations that we present should be considered cautiously and only referred to the reporting of adverse events by hospital workers. In order to minimize biased association, multivariate analyses were conducted, adjusting for the main confounders.

Future studies should be conducted on all COVID-19 vaccines, given that a third dose is currently being recommended in several countries and populations, for rightly collecting adverse reactions to vaccination.

## 5. Conclusions

There is a low frequency of reported adverse reactions to BNT162b2 in hospital workers (4.6%), and all of them were non-severe. Men and physicians tended to underreport their symptoms. The main factors associated with the reported adverse reactions were an antecedent COVID-19 infection and the female sex. The double-dosed regimen was not associated with higher rates of adverse reactions. These data reinforce the safety of this vaccine and should guide future studies regarding the third dose and the adverse reactions to other COVID-19 vaccines.

## Figures and Tables

**Table 1:** Sociodemographic and clinical characteristics stratified by the presence of reported adverse reactions.

Variable	Total (*n* = 3969)	Adverse Reaction (*n* = 182)	No Adverse Reaction (*n* = 3781)	*p*-Value ^1^
	*n* (%)/x (s)	*n* (%)/x (s)	*n* (%)/x (s)	
Age	46.4 (13.9)	48.0 (11.8)	46.3 (14.0)	0.118
Sex				0.027 *
Women	2902 (73.1)	146 (80.2)	2751 (72.8)	
Men	1066 (26.9)	36 (19.8)	1030 (27.2)	
Professional category ^2^				0.010 *
Physician	496 (13.7)	10 (5.9)	485 (14.1)	0.003 *
Healthcare training resident	166 (4.6)	4 (2.4)	161 (4.7)	0.174
Nurse	834 (23.0)	42 (24.9)	791 (22.9)	0.486
Auxiliary nurse	663 (18.3)	33 (19.5)	630 (18.3)	0.604
Warden	303 (8.4)	19 (11.2)	283 (8.2)	0.142
Pregraduate student	49 (1.4)	3 (1.8)	46 (1.3)	0.393
Other healthcare worker	300 (8.3)	12 (7.1)	288 (8.4)	0.610
Administrative worker	242 (6.7)	21 (11.8)	221 (6.2)	0.002 *
Other non-healthcare worker	567 (15.7)	25 (14.8)	542 (15.7)	0.822
Unknown	349 (8.8)	13 (7.1)	334 (8.8)	-
Vaccination regimen				<0.001 *
Double-dosed (completed)	3913 (98.7)	170 (93.4)	3743 (99.0)	
Single-dosed ^3^	50 (1.3)	12 (6.6)	38 (1.0)	
Antecedent of COVID-19 infection ^4^	567 (14.3)	50 (27.6)	517 (13.7)	<0.001 *
Infection before vaccine	452 (11.4)	41 (22.5)	410 (10.8)	<0.001 *
Infection during vaccine	58 (1.5)	5 (2.7)	53 (1.4)	0.126
Infection after vaccine	59 (1.5)	4 (2.2)	54 (1.4)	0.275
Hospitalization	7 (0.2)	2 (4.0)	5 (1.0)	0.124
Serology tests				1.000
Positive IgG after vaccine	1399 (99.6)	68 (100.0)	1331 (99.6)	
Negative IgG after vaccine	6 (0.4)	0 (0.0)	6 (0.4)	
Unknown	2564 (64.6)	114 (62.6)	2444 (54.3)	

^1^ *p*-value of chi-square test or Fisher exact test for all the variables except for age (*t* test). ^2^ The professional category named “other healthcare workers” included physiotherapists, occupational therapists, pharmacists, psychologists, and health technicians such as laboratory technicians and radio-diagnostic technicians, among others. The category “administrative” included administrative personnel, management positions, and clinical documentation and admission technicians. The category “other non-healthcare workers” included security personnel, kitchen workers, maintenance technicians, and cleaning staff. The category “unknown” included workers with missing data regarding the occupational category. ^3^ Professionals who received only one dose for different reasons (e.g., adverse reaction to the first dose, refusal to receive the second dose, etc.). ^4^ Infection during vaccine refers to workers that were infected between the first dose of the vaccine and 7 days after the second dose. Workers who were infected after this period were included in the category “infection after vaccine”. * *p*-value < 0.05.

**Table 2:** Adverse reactions reported by hospital workers after BNT162b2 vaccination.

Adverse Reaction	Total (*n*)	% of the Total Number of Patients Who Reported Adverse Reactions (*n* = 182)	% of the Total Sample of Hospital Workers (*n* = 3969)
General malaise	95	52.2%	2.4%
Fever	92	50.6%	2.3%
Arthromyalgia	80	44.0%	2.0%
Headache	47	25.8%	1.2%
Nausea or vomiting	26	14.3%	0.7%
Dermatologic adverse reactions	26	14.3%	0.7%
Diarrhea	17	9.3%	0.4%
Asthenia	17	9.3%	0.4%
Painful adenopathy	12	6.6%	0.3%
Non-severe neurological symptoms	12	6.6%	0.3%
Neurovegetative symptoms	12	6.6%	0.3%
Catarrhal symptoms	6	3.3%	0.2%
Dyspnea	5	2.7%	0.1%
Vertigo	4	2.2%	0.1%

**Table 3:** Crude and adjusted odds ratio for the report of adverse reactions after COVID-19 vaccination in hospital workers.

Variable	Crude Odds Ratio (95% CI)	Adjusted Odds Ratio (95% CI)	*p*-Value ^1^
Sex (women)	1.52 (1.05–2.20)	1.51 (1.03–2.20)	0.033
Age (per year)	1.01 (1.00–1.01)	1.01 (1.00–1.01)	0.108
Vaccination regimen (single-dosed)	6.95 (3.57–13.55)	4.92 (2.45–9.89)	<0.001
Antecedent of COVID-19 infection	2.40 (1.71–3.37)	2.09 (1.47–2.98)	<0.001
Professional category (physicians)	0.40 (0.21–0.73)	0.41 (0.21–0.80)	0.008

^1^ *p*-value of the adjusted model for the presence of dermatological adverse reactions. The multivariate logistic regression model was adjusted for all variables included in the table. “Men” represents the reference category for sex, “double-dose” represents the reference category for vaccination regimen, “not having an antecedent of COVID-19 infection” is the reference category for this association, and “not being a physician” is the reference category for professional category.

## Data Availability

Datasets will be available from the corresponding author upon reasonable request.
